# Supplementary material for: Modeling glioblastoma heterogeneity as a dynamic network of cell states
Source: Mol Syst Biol. 2021 Sep 16;17(9):e10105. doi: 10.15252/msb.202010105 (PMC8444284; doi:10.15252/msb.202010105)
Supplement: Supplementary file 6 — Source Data for Figure 5 [file MSB-17-e10105-s004.zip › Figure5A_sourcedata/GSEA_3071/hallmarks_stateV.GseaPreranked.1623742546329/HALLMARK_ALLOGRAFT_REJECTION.html]

Details for gene set HALLMARK\_ALLOGRAFT\_REJECTION[GSEA]

|  || Dataset | state43071 |
| Phenotype | NoPhenotypeAvailable |
| Upregulated in class | na\_neg |
| GeneSet | HALLMARK\_ALLOGRAFT\_REJECTION |
| Enrichment Score (ES) | -0.53036994 |
| Normalized Enrichment Score (NES) | -2.5422423 |
| Nominal p-value | 0.0 |
| FDR q-value | 0.0 |
| FWER p-Value | 0.0 |
Table: GSEA Results Summary

  

Fig 1: Enrichment plot: HALLMARK\_ALLOGRAFT\_REJECTION      
 Profile of the Running ES Score & Positions of GeneSet Members on the Rank Ordered List

  

| PROBE | GENE SYMBOL | GENE\_TITLE | RANK IN GENE LIST | RANK METRIC SCORE | RUNNING ES | CORE ENRICHMENT || 1 | BRCA1 |  |  | 46 | 1.592 | 0.0392 | No |
| 2 | AKT1 |  |  | 1477 | 0.486 | -0.2188 | No |
| 3 | APBB1 |  |  | 1668 | 0.442 | -0.2417 | No |
| 4 | TGFB2 |  |  | 1976 | 0.392 | -0.2884 | No |
| 5 | CCND3 |  |  | 1991 | 0.390 | -0.2793 | No |
| 6 | ST8SIA4 |  |  | 2207 | 0.361 | -0.3094 | No |
| 7 | TPD52 |  |  | 2318 | 0.347 | -0.3199 | No |
| 8 | IKBKB |  |  | 2370 | 0.339 | -0.3195 | No |
| 9 | IL7 |  |  | 2500 | 0.322 | -0.3343 | No |
| 10 | CD47 |  |  | 2503 | 0.322 | -0.3250 | No |
| 11 | NCK1 |  |  | 2553 | 0.316 | -0.3248 | No |
| 12 | CCND2 |  |  | 2876 | 0.282 | -0.3777 | No |
| 13 | GLMN |  |  | 3285 | -0.263 | -0.4476 | No |
| 14 | NME1 |  |  | 3615 | -0.317 | -0.5007 | No |
| 15 | TAPBP |  |  | 3712 | -0.333 | -0.5090 | No |
| 16 | TLR3 |  |  | 3825 | -0.354 | -0.5197 | Yes |
| 17 | RIPK2 |  |  | 3867 | -0.362 | -0.5166 | Yes |
| 18 | GZMB |  |  | 3906 | -0.368 | -0.5128 | Yes |
| 19 | ITGB2 |  |  | 3948 | -0.377 | -0.5092 | Yes |
| 20 | TAP2 |  |  | 4017 | -0.392 | -0.5104 | Yes |
| 21 | CTSS |  |  | 4043 | -0.398 | -0.5031 | Yes |
| 22 | BCL10 |  |  | 4078 | -0.404 | -0.4975 | Yes |
| 23 | MTIF2 |  |  | 4110 | -0.411 | -0.4910 | Yes |
| 24 | HIF1A |  |  | 4130 | -0.417 | -0.4820 | Yes |
| 25 | RPS19 |  |  | 4230 | -0.449 | -0.4874 | Yes |
| 26 | ABCE1 |  |  | 4239 | -0.452 | -0.4753 | Yes |
| 27 | IFNGR1 |  |  | 4240 | -0.452 | -0.4617 | Yes |
| 28 | RPS3A |  |  | 4266 | -0.462 | -0.4525 | Yes |
| 29 | CDKN2A |  |  | 4301 | -0.472 | -0.4448 | Yes |
| 30 | BCAT1 |  |  | 4314 | -0.476 | -0.4327 | Yes |
| 31 | F2R |  |  | 4316 | -0.476 | -0.4185 | Yes |
| 32 | RPL39 |  |  | 4323 | -0.478 | -0.4053 | Yes |
| 33 | B2M |  |  | 4341 | -0.485 | -0.3939 | Yes |
| 34 | IL15 |  |  | 4346 | -0.486 | -0.3800 | Yes |
| 35 | RPL9 |  |  | 4506 | -0.544 | -0.3939 | Yes |
| 36 | PSMB10 |  |  | 4628 | -0.608 | -0.3987 | Yes |
| 37 | EIF3D |  |  | 4676 | -0.631 | -0.3887 | Yes |
| 38 | CAPG |  |  | 4709 | -0.650 | -0.3752 | Yes |
| 39 | EIF3J |  |  | 4741 | -0.671 | -0.3609 | Yes |
| 40 | EIF5A |  |  | 4746 | -0.676 | -0.3413 | Yes |
| 41 | THY1 |  |  | 4874 | -0.781 | -0.3419 | Yes |
| 42 | LIF |  |  | 4882 | -0.789 | -0.3195 | Yes |
| 43 | TGFB1 |  |  | 4944 | -0.861 | -0.3052 | Yes |
| 44 | FLNA |  |  | 4948 | -0.862 | -0.2798 | Yes |
| 45 | NPM1 |  |  | 5040 | -1.017 | -0.2665 | Yes |
| 46 | TIMP1 |  |  | 5134 | -1.251 | -0.2465 | Yes |
| 47 | IL11 |  |  | 5170 | -1.414 | -0.2106 | Yes |
| 48 | SOCS1 |  |  | 5184 | -1.475 | -0.1686 | Yes |
| 49 | EGFR |  |  | 5230 | -1.765 | -0.1240 | Yes |
| 50 | IL18 |  |  | 5237 | -1.816 | -0.0704 | Yes |
| 51 | INHBA |  |  | 5282 | -2.697 | 0.0025 | Yes |
Table: GSEA details [plain text format]

  

Fig 2: HALLMARK\_ALLOGRAFT\_REJECTION: Random ES distribution      
 Gene set null distribution of ES for **HALLMARK\_ALLOGRAFT\_REJECTION**
